# Children in out of home care in the youth justice system – understanding when they may be more vulnerable to further offending.

**DOI:** 10.23889/ijpds.v7i3.2031

**Published:** 2022-08-25

**Authors:** Helen Hodges, Kevin Fahey

**Affiliations:** 1 CASCADE; 2 Cardiff University; 3 University of Nottingham

## Objectives

By linking external data to that already held in SAIL Databank, a fuller picture of the sometimes-chaotic lives of those in the youth justice system has been compiled. By looking at the impact of changes in circumstances on the likelihood of offending, opportunities for intervention have been identified.

## Approach

Data from two Welsh youth justice services are used to model how the probability of further offending changes over time for different groups. By linking data from the risk assessment process previously used across England & Wales, to children’s’ social services, education and health records within SAIL Databank, we can for the first-time model their needs and circumstances, and establish temporal precedence in relation to offending. These linked records create an original longitudinal dataset to undertake within- and between- group analysis with a particular focus on understanding the complex relationship between risk and protective factors, and offending for minority groups.

## Results

Adopting an intersectional approach, multi-level modelling has been undertaken to ascertain how the likelihood of further offending changes in response to changes in circumstances (e.g., change of school/placement, starting or finishing treatment for substance misuse or mental health issues) and in response to events linked to the administration of justice (breaches, court appearances and time in custody/remand). In seeking to understand the drivers of recidivism and desistence for different groups of young people, a range of advanced statistical techniques are adopted to most effectively leverage these novel data. Key findings from this research support the need for take up of approaches tailored to the needs and circumstances of the individual.

## Conclusion

Our ongoing research, in collaboration with key stakeholders, will help improve the outcomes of those young people who have come into conflict with the law. Such research will identify where there is need for timely, appropriate multi-agency support tailored to the needs and circumstances of the individual.

